# Supplementary material for: Genetic and Molecular Interactions between HΔCT, a Novel Allele of the Notch Antagonist Hairless, and the Histone Chaperone Asf1 in Drosophila melanogaster
Source: Genes (Basel). 2023 Jan 13;14(1):205. doi: 10.3390/genes14010205 (PMC9858708; doi:10.3390/genes14010205)
Supplement: Supplementary file 1 [file genes-14-00205-s001.zip › genes-2120409-supplementary.pdf]

Supplement: Maier et al.

Genetic and molecular interactions between *$H^{\Delta CT}$*, a novel allele of the Notch antagonist Hairless, and the histone chaperone Asf1 in *Drosophila melanogaster*

contains six Figures S1-S6

S1 **Overexpression of H and $H^{\Delta CT}$**

S2 **In vivo *$H^{\Delta CT}$* protein expression**

S3 **Small eyes induced by ectopic Asf1 expression are rescued by a loss of H activity**

S4 **Eye-specific downregulation of Asf1 results in smaller eyes**

S5 **Asf1 is extremely well conserved in eukaryotes from yeast to fly**

S6 **Secondary structure prediction for NTCT**

S1 Figure Overexpression of H and H$^{\Delta CT}$

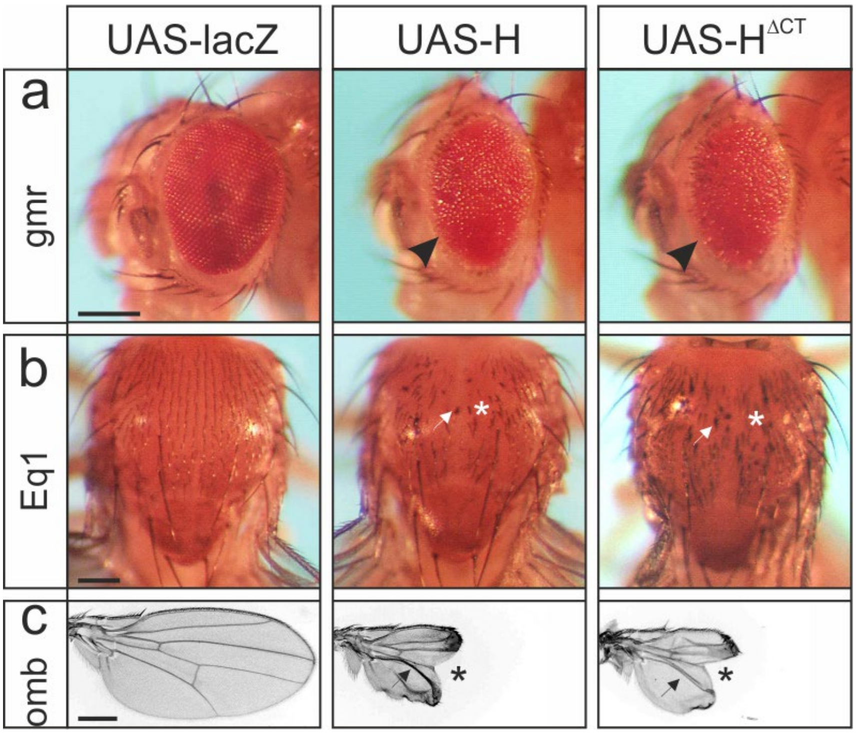

Tissue specific overexpression of UAS-lacZ for control, UAS-H and UAS-H$^{\Delta CT}$ using the Gal4/UAS-system. **(a)** Overexpression of UAS-H, and likewise of UAS-H$^{\Delta CT}$ during retinal development using gmr-Gal4 results in smaller eyes with rough appearance due to apoptosis (arrowhead). Size bar, 200 µm. **(b)** Induction of UAS-H in the central domain of the developing thorax using Eq1-Gal4 affects bristle development. Sensory organs are lacking due to increased lateral inhibition causing baldness (asterisk marks example). In addition, bristle tufts are observed due to a transformation of sockets into shafts (arrow points to example). These phenotypes are also detected in the UAS-H$^{\Delta CT}$ overexpressing flies. Size bar, 200 µm. **(c)** The phenotypes arising from the overexpression of UAS-H and likewise of UAS-H$^{\Delta CT}$ in the central portion of the wing anlagen using omb-Gal4 are characterized by a remarkable tissue loss (asterisk) and thickening of longitudinal veins (arrow points to example). Size bar, 500 µm.

S2 Figure In vivo $H^{\Delta CT}$ protein expression

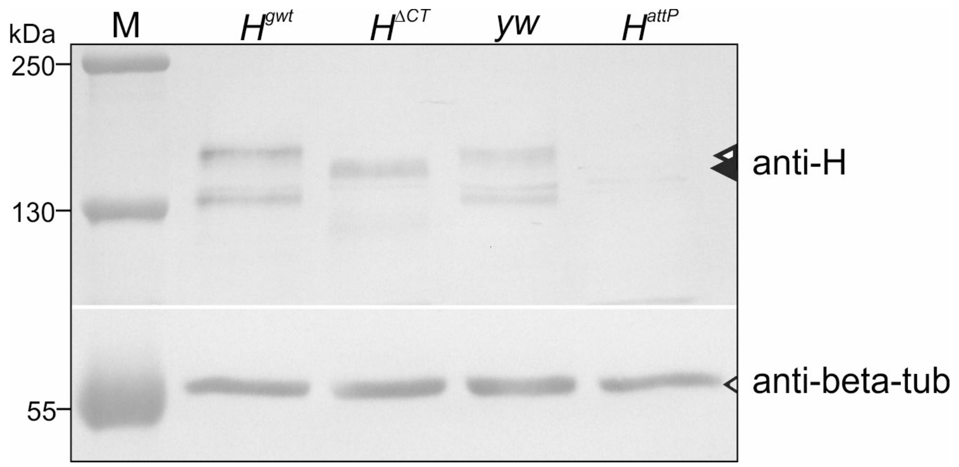

Protein extracts from third instar larval imaginal discs. Homozygotes for the wild type controls H^{gwt} and y^1 w^{67c23} (yw), for $H^{\Delta CT}$ as well as for H^{attP} null mutants as negative control were probed in Western blots for H protein expression using anti-H antibodies. The upper H^{p150} protein band is marked by a white arrowhead. Note slightly reduced size of $H^{\Delta CT}$ protein (black arrowhead). Endogenous beta-tubulin served as loading control (open arrowhead). M, protein standard, size is indicated in kDa.

S3 Small eyes induced by ectopic Asf1 expression are rescued by a loss of H activity

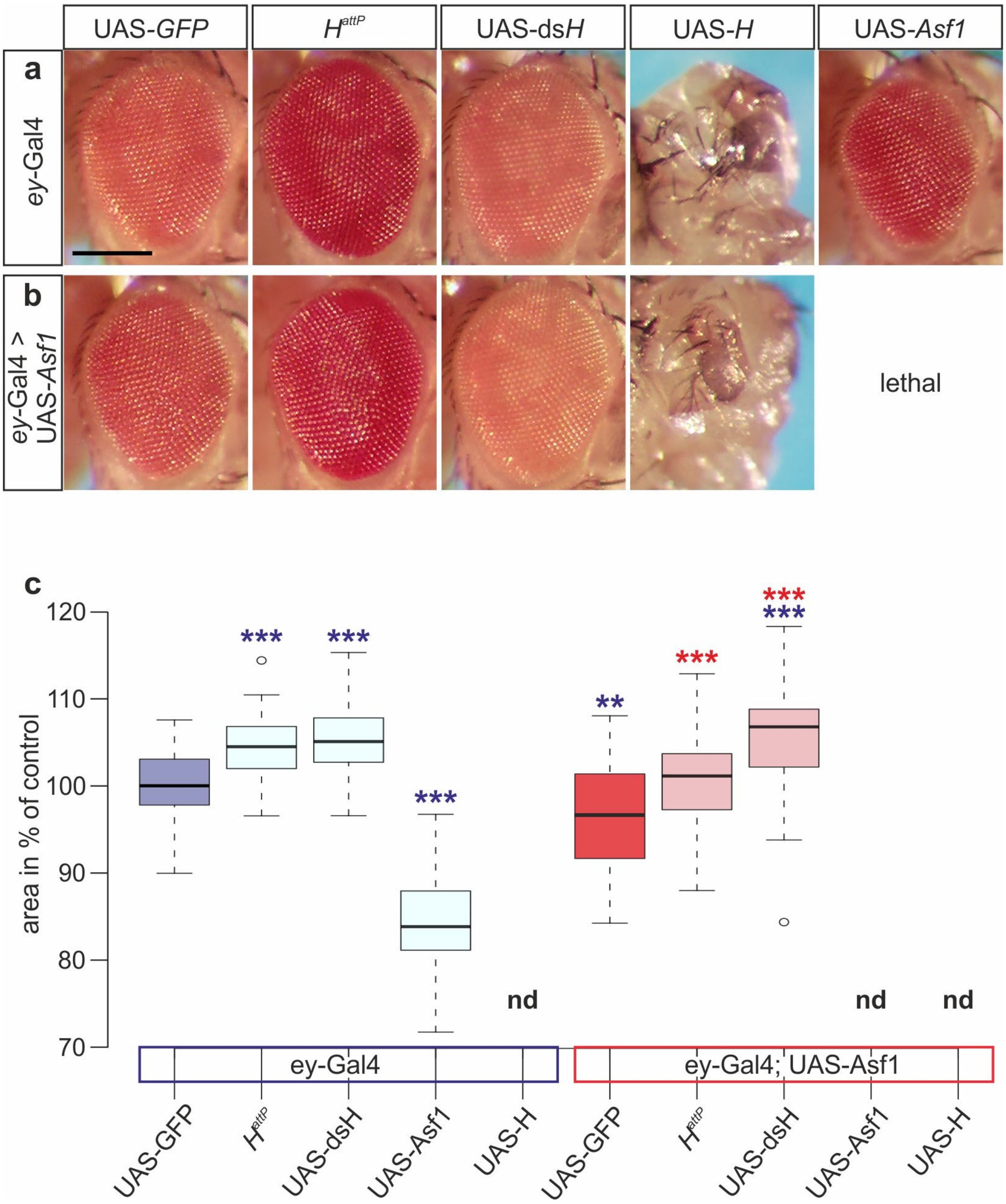

(a) Representative eyes from female flies derived from a cross of ey-Gal4 with the given strains. Note absence of eyes upon the overexpression of H; animals die as pharate adults and were dissected from the pupal case. **(b)** The respective cross performed with an ey-Gal4 UAS-Asf1 strain. The homozygotes are lethal. Size bar 200 µm in (a,b). **(c)** Quantification of female eye size from (a, blue color) and (b, red color) is shown. Box blot limits indicate 25th and 75th percentiles; whiskers extend 1.5 time the interquartile range; center lines show the medians, outliers are indicated by dots; n=40 (assembled by BoxPlotR). Significance was determined with ANOVA two-tailed Tukey-Kramer's test for multiple comparisons; significant differences are indicated relative to the GFP-control (***p≤0.001; **p≤0.01).

S4 Eye-specific downregulation of Asf1 activity results in smaller eyes

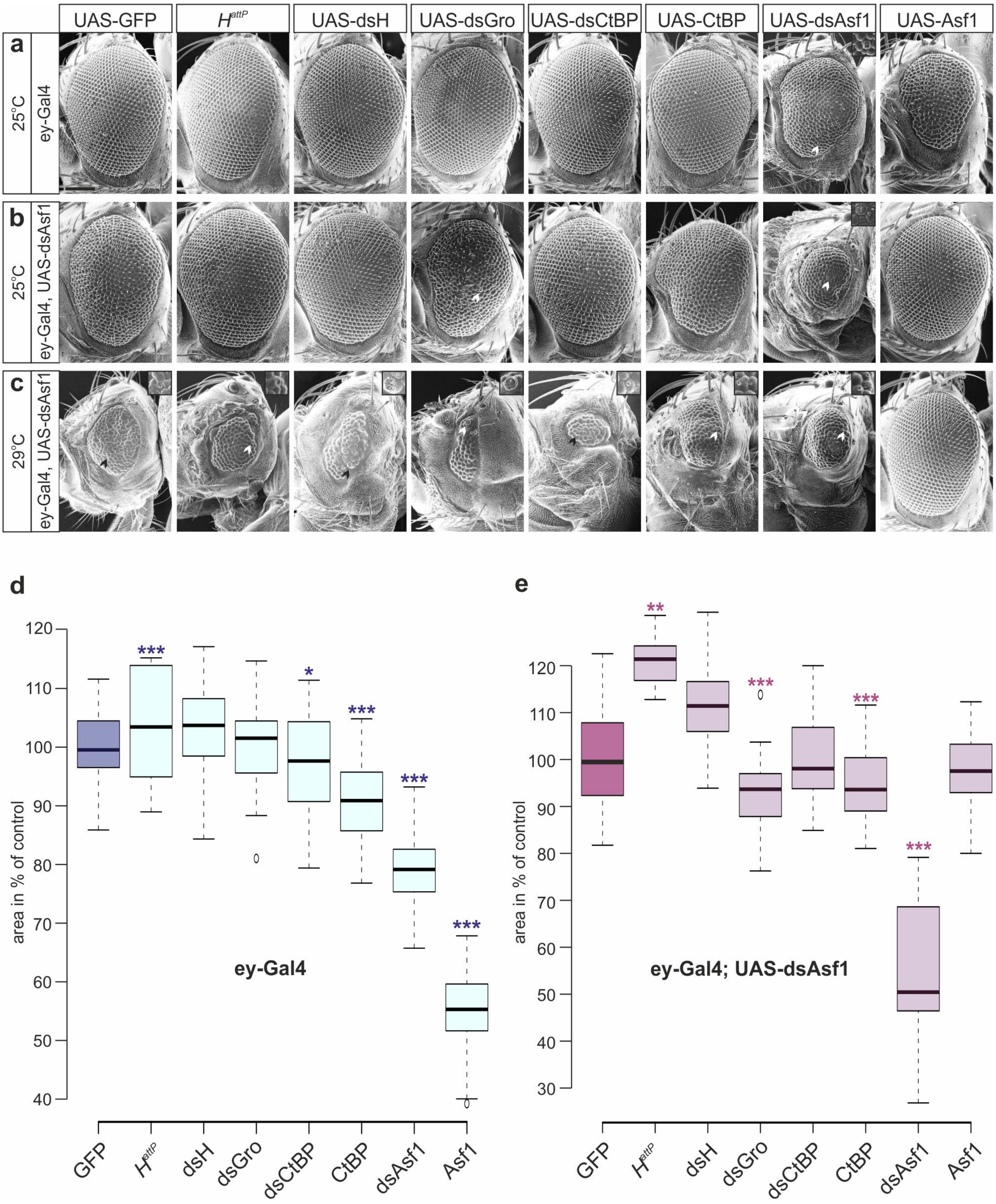

(a-c) Scanning electron micrographs of female eyes. (**a**) Offspring derived from a cross of flies with the given genotypes with ey-Gal4. Note smaller eyes with rough appearance resulting from either downregulation of Asf1 activity by RNA interference (dsAsf1) and by ectopic expression of Asf1. The overexpression or downregulation of either H, Gro or CtBP had little effect on the overall appearance. Note however, that overexpression of H and Gro with ey-Gal4 induced lethality. **(b,c)** Eye-specific expression of Asf1 in combination with the given genotypes at 25°C and 29°C. Temperature sensitivity of the Gal4 system caused stronger phenotypes at 29°C. Due to UAS-titration effect, the phenotype of

ey-Gal4 UAS-Asf1 combined with UAS-GFP is markedly weaker than that of ey-Gal4 UAS-Asf1 alone (compare with a). Blueberry type facets (arrowheads point to examples), indicative of cell death, is shown in enlargements in (b,c). Note enhancement of the small eye phenotype by a downregulation of Gro and of CtBP, as well as a rescue by a downregulation of H or by the concurrent overexpression of Asf1. Extreme phenotypes at 29°C do not allow further evaluation apart from the Asf1 rescue. Size bar 100 µm in (a-c). **(d,e)** Quantification of eye size from the above experiments. Box plots were generated using BoxPlotR. Center lines show the medians; box limits indicate the 25th and 75th percentiles as determined by R software; whiskers extend 1.5 times the interquartile range; outliers are represented by dots. **(d)** n= 29, 6, 29, 29, 29, 29, 31, 29 sample points; **(e)** n = 30, 14, 30, 30, 30, 18, 13, 30 sample points. Significance was determined by ANOVA two-tailed Tukey-Kramer's test for multiple comparisons; significant differences are indicated relative to the respective GFP-control (***p≤0.001; **p≤0.01; *p≤0.05).

S5 Asf1 is extremely well conserved in eukaryotes from yeast to fly

a

```
Dm   1     MAKVHITNVVVLDNPSSFFNPFQFELTFECIEELKEDLEWKMIYVGSAESEEHDQVLDTIYVGPVPEGRHIFVFQADPPD  80
Sc   1     MSIVSLLGIKVLNNPAKFTDPYEFEITFECLESLKHDLEWKLTYVGSSRSLDHDQELDSILVGPVPVGVNKFVFSADPPS  80

Dm   81    VSKIPEPDAVGVTIVLLTCSYRGQEFVRVGYYVNNDYADPEMRENPPTKPLFEKLTRNILASKPRVTRFKINWDYGHING  160
Sc   81    AELIPASELVSVTVILLSCSYDGREFVRVGYYVNNEYDEEELRENPPAKVQVDHIVRNILAEKPRVTRFNIVWD----NE  156

Dm   161   N-GN-------GV--------------ENGHQDEM--ATDGPSTSEAASAVIHPED----DNSLAMP-----MEN-----  202
Sc   157   NEGDLYPPEQPGVDDEEEEDDEEEDDDEDDEDDEDDDQEDGEGEAEEAAEEEEEEEEKTEDNETNLEEEEEDIENSDGDE  236

Dm   203   --GIKAL-----NENSN----------SLAMEC----------  218
Sc   237   EEGEEEVGSVDKNEDGNDKKRRKIEGGSTDIESTPKDAARSTN  279
```

b

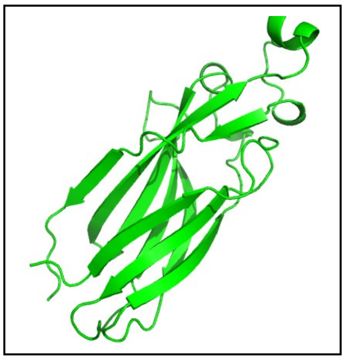

c

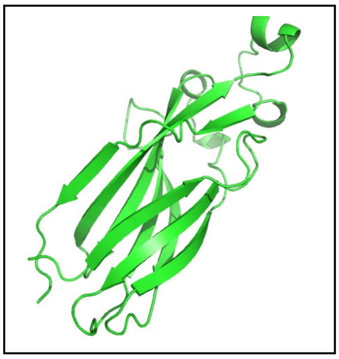

(a) Comparison of primary sequence of Asf1 from *Drosophila melanogaster* (Dm; NP_001189131.1) and yeast *Saccharomyces cerevisiae* (Sc, NP_012410.1). Identical amino acids are shown in red, similar ones in blue. High conservation of 59% identity is observed in the histone chaperone domain located in the N-terminal 1-154 amino acids. **(b)** Structure of *D. melanogaster* Asf1 protein in ribbon diagram (PDB-ID 2IO5). **(c)** Structure of *S. cerevisiae* ASF1 (PDB-ID 1roc).

S6 Secondary structure prediction for NTCT

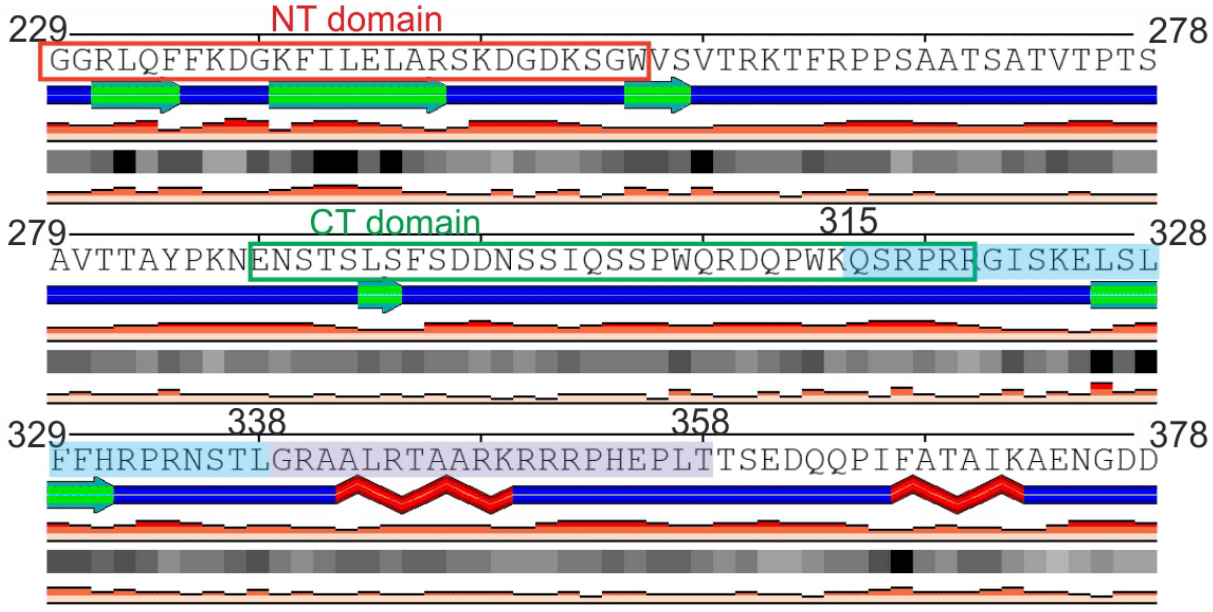

In silico prediction for NTCT's secondary structure using POLYVIEW-MM (http://polyview.cchmc.org/conform.html; accessed 01.12.2022). Numbers correspond to H codons. Blue line, unstructured loop or coil; green, beta strand; red, alpha helix. Below, indicator for variability: the more red, the more flexible. Grey reflects relative solvent accessibility: the brighter, the more surface exposed. Frequency of contacts indicated by height and red color. Boxed lines: NT domain (red) and CT domain (green), corresponding to highly conserved regions are indicated. The colored region 315-358 is minimally required for Asf1 binding; neither 232-338, nor 339-358 are sufficient. They contain a predicted beta strand and an alpha helix, respectively.
